# Thermal characteristics of magnetized hybrid Casson nanofluid flow in a converging–diverging channel with radiative heat transfer: a computational analysis

**DOI:** 10.1038/s41598-023-49397-3

**Published:** 2023-12-11

**Authors:** Noreen Sher Akbar, M. Fiaz Hussain, Metib Alghamdi, Taseer Muhammad

**Affiliations:** 1 DBS&H, CEME, National University of Sciences and Technology, Islamabad, Pakistan; 2 Department of Mathematics, COMSATS University Islamabad, Vihari Campus, Islamabad, 61100 Pakistan; 3 Department of Mathematics, College of Science, King Khalid University, 61413 Abha, Saudi Arabia

**Keywords:** Mathematics and computing, Nanoscience and technology

## Abstract

In the present article we consider the physical model of two-dimensional Casson hybrid nanofluids flow, which is magnetized and thermally radiative, laminar, incompressible inside the channel. Flow equations have been modelled for two dimensional axial and radial velocity components $u$ along $x{\text{-axis}}$ and $v$ along the $y{\text{-axis}}$. There exists temperature $\left({T}_{1}>{T}_{2}\right)$ which is constant for upper and lower walls. The Casson nanofluids model with nano type particles includes heat transfer effect between two stretched and shrinking walls of the channel was constructed. The continuity, momentum and energy equations are modelled in cartesian coordinates system. The finite element technique is used to evaluate numerical solutions for velocity, temperature, Skin friction and Nusselt number. It is evident that the hybrid Casson nanofluids exhibit opposite behaviors in the stretching and shrinking cases near the upper and lower walls of the channel. It is also observed that in the stretching case, increasing the values of the Casson parameter leads to a rise in both shear stress and heat transfer rate for both plates of the channel. However, the results contradict this trend in the shrinking case. Understanding the thermal characteristics of magnetized hybrid fluids can be applied to the design of advanced cooling systems in engineering applications, biomedical fluid dynamic, in energy system this study can be applied to improve the efficiency of energy systems where fluid flow and heat transfer play crucial roles. Further use of nanofluids suggests a connection to nanotechnology, and the study may have implications for the development of advanced nanomaterial-based heat transfer fluids.

## Introduction

Non-Newtonian fluids, which behave differently from Newtonian fluids, have been studied since the nineteenth century. Their behavior depends on factors like shear rate, pressure, and temperature, unlike Newtonian fluids with constant viscosity. Casson fluids, a subset of non-Newtonian fluids, display characteristics of both solids and liquids. They derive their name from Sydney Goldstein Casson^1^, a British scientist who introduced their behavior in his paper “Flow of clay water pests” 1959. However, the understanding and exploration of non-Newtonian fluids and their diverse flow behaviors predate Casson's research. In the early twentieth century, the study of non-Newtonian fluids experienced notable progress and interest. Key contributions were made by scientists such as Paul Weissenberg and Eugene Bingham, who significantly advanced the understanding of fluid rheology, focusing on the flow and deformation of materials. The utilization of nanoparticles in Casson fluids to form nanofluids has emerged as a relatively recent advancement, coinciding with the growing prominence of nanofluid research in the late 20th and early twenty-first centuries. The term "Casson nanofluids" likely originated as scientists investigated the fusion of Casson fluid characteristics with the integration of nanoparticles, leading to the adoption of this specific terminology.

Casson nanofluids are nanofluids with the rheological properties defined by the Casson model. Nanofluids are manufactured fluids that include nanoparticles distributed in a base fluid, such as water or oil. These nanoparticles, which can be metallic, ceramic, or carbon-based, provide unique qualities to the base fluid, improving thermal conductivity, heat transfer characteristics, and other desired features. Casson nanofluids have opened up new avenues for improving heat transfer and fluid flow applications. Casson nanofluids can outperform typical fluids in terms of heat conductivity by introducing nanoparticles into the base fluid. The improved thermal conductivity enables more effective heat dissipation and transmission in a variety of systems and devices. Fluids in the human body include blood, synovial fluid, mucus, and cerebrospinal fluid, among others. These fluids have varying rheological properties and are frequently explained using other models, such as Newtonian or non-Newtonian fluid models. Blood, for example, is a complicated fluid with non-Newtonian behavior. It is a shear-thinning fluid, which means that its viscosity lowers with increasing shear rate. This feature makes blood flow more readily through blood arteries, lowering resistance. The hematocrit (the proportion of red blood cells in the blood) and plasma proteins have the greatest impact on blood viscosity.

For this era Flow of a Casson fluid between two rotating cylinders proposed by Batra and Das^2^. Further, Flow of Casson nanofluid with viscous dissipation and convective conditions: A mathematical model by Hussain et al.^3^. The objective of this study is to investigate the similarity solution for the steady boundary layer flow and heat transfer of a Casson nanofluid over a vertical cylinder. The cylinder undergoes exponential stretching in the radial direction MAlik et al.^4^ described in his article the boundary layer flow of Casson nanofluid over a vertical exponentially stretching cylinder. Analytical modeling of entropy generation for Casson nano fluid flow induced by a stretching surface by Aboalbashri et al.^5^.

### Literature survey

For both slip and no-slip conditions, the hybrid nanofluid velocity shows an upward trend for both the stretching and mixed convection parameters graphically, after their numerical simulation in his article^6^. Numerical studies of MHD flow in Casson nanofluid combined with Joule heating and slip boundary conditions^7^. Recently^8^ analytical analysis of the magnetic field, heat generation and absorption, viscous dissipation on couple stress casson hybrid nano fluid over a nonlinear stretching surface. The phenomenon of heat enhancement in hybrid nanofluid flow through the peristaltic mechanism has garnered significant interest due to its relevance in various engineering and biomedical systems. Examples include flow through canals, the cavity flow model, and applications in biomedicine introduce^9^. Theory explores the dynamic behavior of a hybrid Casson nanofluid with laser radiation and chemical reaction as it flows through sinusoidal channels. The study focuses on understanding the complex interplay between these factors and their impact on the fluid flow and heat transfer characteristics^10^. The study modeled activation energy and chemical reaction on non-Newtonian liquid motion on a stretching sheet. It used numerical techniques and MATLAB to analyze the system and presented comparative results of linear and nonlinear stretching sheet effects^11^. The study proposed by^12^ examines entropy generation in a magnetohydrodynamic flow of a hybrid Casson nanoliquid in a porous channel. Numerical analysis shows that a magnetic field increases entropy generation, while porous media decreases it. Heat transfer rate decreases away from the lower wall in the channel. Numerical simulation of nonlinear thermal radiation on the 3D flow of a couple stresses Casson nanofluid due to a stretching sheet give by^13^. In the^14^ research, the modeling of pulsatile blood flow is carried out using the Casson fluid model in an overlapping stenotic artery. The study also incorporates Au-Cu hybrid nanoparticles and employs a varying viscosity approach. This article^15^ examines MHD boundary layer Casson hybrid nanofluid flow and heat transfer around an exponentially stretched cylinder with a heat source. The results show higher thermal conductivity and improved heat transfer for the Casson hybrid nanofluid compared to Casson nanofluid. Skin friction coefficient increases by up to 29% for the hybrid nanofluid. Hall and ion slip effects on the MHD flow of Casson hybrid nanofluid past an infinite exponentially accelerated vertical porous surface presents by^16^. The study examines a blood-based hybrid nanofluid with carbon nanotubes (CNTs) over a stretching sheet. It considers a perpendicular magnetic field and explores the effectiveness of CNTs in the carrier fluid for various applications^17^. Thermal performance of iron oxide and copper $({{\text{Fe}}}_{3}{{\text{O}}}_{4},\mathrm{ Cu})$ in hybrid nanofluid flow of Casson material with Hall current via complex wavy channel model represents by^18^. This study investigates^19^ heat and mass transfer in the boundary layer flow of chemically reactive Casson nanoparticles (Ag and MgO) with a magnetic dipole and gyrotactic microorganism over a stretching cylinder. Generalized Fourier and Fick laws, as well as thermal radiation effects, are considered for energy transportation. The inclusion of nanoparticles aims to enhance heat transport and fluid thermal conductivity. The flow equations are transformed into nonlinear coupled ODEs and solved using the Bvp4c MATLAB approach. Graphical and tabulated data show the effects of parameters on velocity, concentration, temperature, microorganism density, microorganism transport rate, and skin friction coefficient. Results indicate that increasing the curvature parameter α boosts fluid velocity, while stronger slip and magnetic parameters decrease it.^20^ Work on entropy generation on biomagnetic gold-copper/blood hybrid nanofluid flow driven by electro-kinetic force in a horizontal irregular channel with bioconvection phenomenon.

Zaheer et al.^21^ find the analytical solution on radiative MHD Casson nanofluid flow with activation energy and chemical reaction over past nonlinearly stretching surface through Entropy generation. After that Numerical analysis of Casson nanofluid three-dimensional flow over a rotating frame exposed to a prescribed heat flux with viscous heating proposed by Wael and Wahib^22^. Reynolds nano fluid model for Casson fluid flow conveying exponential nanoparticles through a slandering sheet represented by Sohail et al.^23^. A valid numerical solution and heat transfer enhanced effect find by Suresh et al.^24^ in his recent article numerical analysis of magneto hydrodynamics Casson nanofluid flow with activation energy with Hall current and thermal radiation. Further recent literature to nanofluid includes.^32–45^.

### Studies for primitive

Currently, hybrid and ternary hybrid nanofluids are extensively utilized for research purposes. Numerous researchers have recently published articles on Non-Newtonian flow using hybrid-type nanofluids in channels over the past few years. In our present study, we obtained relevant findings by employing similar variables. The motivation behind these results comes from the previously published articles mentioned below.

- The present study focuses on hybrid Casson nanofluids' flow into a channel, drawing significant inspiration from the works of the authors^25^ and^26^. They have extensively discussed numerical fluid flow problems and elaborated on the heat transfer effects, encompassing non-Newtonian Casson fluids combined with nanoparticles.
- Qadeer et al.^27^ represents the mathematical modeling of nanolayer on biological fluids flow through porous surfaces in the presence of CONTs. They make use of the findings pertaining to the Casson fluid flow in a channel, considering the influence of nanolayer thermal conductivity.
- Optimization of MHD flow of radiative micropolar nanofluid in a channel by RSM (Sensitivity Analysis) proposed by Reham et al.^28^. They model the stretching and shrinking behavior of the channel using Blood fluids as a single-phase model. Additionally, they incorporate thermal radiation to investigate the heat transfer effects of the non-Newtonian fluid.

The research mentioned above is primarily a literature review, providing motivation and context for our current studies. It is essential to comprehend the structure of hybrid Casson nanofluids flowing in channels. However, the author has not conducted any research on hybrid Casson nanofluids in channels involving two stretching and shrinking cases. Further research on non-Newtonian fluids is warranted to explore their heat transfer behavior, especially when considering the effects of thermal radiation and magnetized nanoparticles.

### Modulation structure

The following axioms are the basic structure of current research.

- The two phase model (Buongiorno's model) was adapted and modified to accommodate hybrid Casson nanofluids as non-Newtonian fluids flowing into a channel. In this study, appropriate boundary conditions were proposed for two cases of stretching and shrinking.
- Thermal radiations and Casson parameter are also introduced into the channel to enhance the appeal of the problem concerning heat transfer and flow of the blood.
- The most optimal numerical techniques are employed to ensure solution convergence in accordance with the modeled stretching and shrinking cases.

### Applications in biomedical fields

The utilization of hybrid Casson nanofluids in a channel involves examining the behavior of flow and heat transfer properties within the channel. These nanofluids, formed by combining Casson fluids with nanoparticles, possess distinctive characteristics that make them attractive for a range of engineering applications, particularly in heat transfer systems. Researchers investigate their interaction with channel boundaries, the impact on heat transfer rates, and their response to varying flow conditions. Comprehending their behavior in channels can pave the way for advancements in fields like cooling systems, heat exchangers, and thermal management applications.

## Mathematical formulation

**Figure 1:**
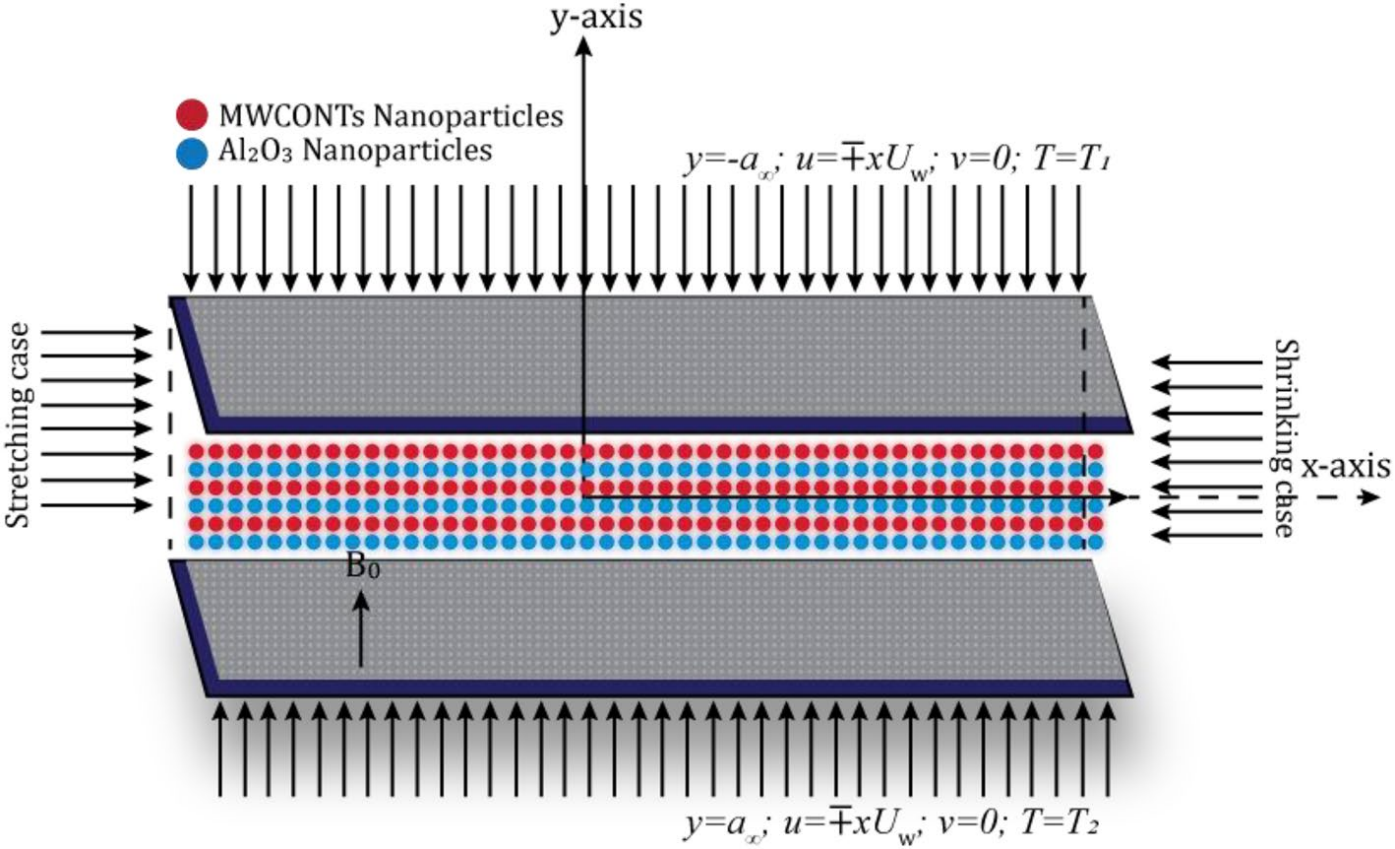
Physical modal of channel.

By ignoring the other reacted terms such as gravitational field and some frictional forces, the casson nanofluids model with nano type particles $({{\text{Al}}}_{2}{{\text{O}}}_{3}-{\text{MW}}/{\text{CONTs}})$ includes heat transfer effect between two stretched and shrinking walls of the channel was constructed. The fluid is thought to be a non-Newtonian, time-independent, laminar inside the 2D $R\left(z\right)=(x,y,0)$ permeable channel with the stretch and shrink walls of total length $\gamma =2{a}_{[-\infty , \infty ]}$. The existing physical model demonstrates two scenarios: stretching and shrinking, with respect to the reference velocity ${U}_{w}$. If ${U}_{w}$ is negative, the region is observed to stretch, whereas if ${U}_{w}$ is positive, the region is seen to shrink, as illustrated in Fig. 1.

### Fundamental equation of fluids dynamics

Let we take Navier–Stokes equations of two phase modal having two dimensional axial and radial velocity components $u$ along $x-axis$ and $v$ along the $y-axis$.

1 $$\frac{\partial u}{\partial x}+\frac{\partial v}{\partial y}=0,$$

2 $${\rho }_{hbf}\left(u\frac{\partial u}{\partial x}+v\frac{\partial u}{\partial y}\right)+\frac{\partial P}{\partial x}={\mu }_{hbf}\left(1+\frac{1}{{\beta }_{c}}\right)\left(\frac{{\partial }^{2}u}{\partial {x}^{2}}+\frac{{\partial }^{2}u}{\partial {y}^{2}}\right)+{\sigma }_{s}{B}_{0}^{2}u,$$

3 $${\rho }_{hbf}\left(u\frac{\partial v}{\partial x}+v\frac{\partial v}{\partial y}\right)+\frac{\partial P}{\partial y}={\mu }_{hbf}\left(1+\frac{1}{{\beta }_{c}}\right)\left(\frac{{\partial }^{2}v}{\partial {x}^{2}}+\frac{{\partial }^{2}v}{\partial {y}^{2}}\right),$$

4 $$({\rho Cp)}_{hbf}\left(u\frac{\partial T}{\partial x}+v\frac{\partial T}{\partial y}\right)={K}_{hbf}\frac{{\partial }^{2}u}{\partial {y}^{2}}-\frac{\partial {q}_{\xi }}{\partial y}+\tau ({\rho Cp)}_{hbf}\left(\frac{{D}_{B}{\chi }_{c}}{a}\frac{\partial T}{\partial y}+\frac{{D}_{T}}{{T}_{2}} {\left(\frac{\partial T}{\partial y}\right)}^{2}\right)+{\mu }_{hbf}{u}^{2}.$$

Subjected to the boundaries,

5 $$\begin{aligned} &u=\mp x{U}_{w}, v=0, T={T}_{1} \,at \,upper \,plate \left(y=-{a}_{\infty }\right),\\ & u=\mp x{U}_{w}, v=0, T={T}_{2} \,at \,lower \,plate \left(y={a}_{\infty }\right). \end{aligned}$$

Here $P$ stands for pressure ${\beta }_{c}$ shows casson parameter, ${\sigma }_{s}$ and ${B}_{0}^{2}$ denoted the electric and field of Casson nanofluid inside the walls of channel. Where $\mu , \rho , K, (\rho CP)$ with subscripts $hbf$ shows the dynamic viscosity, density, thermal conductivity and heat capacitance of casson nanofluids with nanoparticles $({{\text{Al}}}_{2}{{\text{O}}}_{3}-{\text{MW}}/{\text{CONTs}})$ but the subscripts $bsf$ shows the same as Thermophysical properties of based fluids which is blood (casson fluid). The Thermophysical properties of hybrid Casson nanofluids shows in Tables 1 and 2^29,30^.

**Table 1:** Thermophysical properties^29,30^ of hybrid Casson nanofluids $(hbf)$,$(MWCONTs-A{l}_{2}{O}_{3})/Blood.$

Properties	$({\text{cu}}-{{\text{Al}}}_{2}{{\text{O}}}_{3})/{{\text{H}}}_{2}{\text{O}}$ (hbf)
Dynamic viscosity $(\mu )$	${\mu }_{hbf}={\mu }_{bsf}{\left(\left(1-{\phi }_{p1}\right)\left(1-{\phi }_{p2}\right)\right)}^{-2.5}$
Density $(\rho )$	${\rho }_{hbf}={\rho }_{bsf}\left(1-{\phi }_{hdf}+{\phi }_{p1}\frac{{\rho }_{p1}}{{\rho }_{bsf}}+{\phi }_{p2}\frac{{\rho }_{p2}}{{\rho }_{bsf}}\right)$
Heat capacitance $({C}_{p})$	${(\rho {C}_{p})}_{hbf}={(\rho CP)}_{bsf}\left(1-{\phi }_{hdf}+{\phi }_{p1}\frac{({\rho CP)}_{p1}}{{(\rho CP)}_{bsf}}+{\phi }_{p2}\frac{{(\rho CP)}_{p2}}{({\rho CP)}_{bsf}}\right)$
Thermal conductivity $({\text{K}})$	${K}_{hbf}={K}_{bsf}\left(\frac{\frac{{\phi }_{p1}{K}_{p1}+{\phi }_{p2}{K}_{p2}}{{\phi }_{hbf}}+2{K}_{bsf}+2\left({\phi }_{p1}{K}_{p1}+{\phi }_{p2}{K}_{p2}\right)-2{\phi }_{hbf}{K}_{bsf}}{\frac{{\phi }_{p1}{K}_{p1}+{\phi }_{p2}{K}_{p2}}{{\phi }_{hbf}}+2{K}_{bsf}-\left({\phi }_{p1}{K}_{p1}+{\phi }_{p2}{K}_{p2}\right)+{\phi }_{hbf}{K}_{bsf}}\right)$

**Table 2:** Thermophysical properties^32^ of blood and nanoparticles.

Expressions	$\rho \left({\text{kg}}/{{\text{m}}}^{3}\right)$	${C}_{p }({\text{J}}/{\text{kgk}})$	$\kappa ({\text{w}}/{\text{mk}})$
$Blood(bsf)$	1053	3594	0.492
$A{l}_{2}{O}_{3}(p1)$	8933	385	400
$MW/COTs(p2)$	1600	796	3000

There are ${\phi }_{p1}$ and ${\phi }_{p2}$ shows the volume fraction of two nanoparticles which are cupper $(MWCONTs)$ and alumina $({{\text{Al}}}_{2}{{\text{O}}}_{3})$. So, the volume fraction of hybrid nanofluids is ${\phi }_{hbf}={\phi }_{p1}+{\phi }_{p2}$. Other side the fixed values of ${\rho }_{p1}, {\rho }_{p2},({\rho CP)}_{p1},({\rho CP)}_{p2},{K}_{p1},{K}_{p2}$ are density, heat capacitance and thermal conductivity of both type nanoparticles.

To modify the temperature equation we considered the Rosceland approximation of radiation^31^,

6 $${q}_{\xi }=-\frac{16{\sigma }^{*}{T}^{3}}{3{k}^{*}}.\frac{\partial {T}^{4}}{\partial y},$$

Here, ${\sigma }^{*}$ represents the Stefan-Boltzmann constant, valued at $(5.6729\times {10}^{-8}\mathrm{ W}/{{\text{m}}}^{2}{{\text{k}}}^{4})$. The term ${k}^{*}$ denotes the radiation impact of a black body with thermal emission, further calculated by the emissive body radiation effect through the product of the Stefan-Boltzmann constant and absolute temperature, given by ${\sigma }_{eb} = {\sigma }^{*}{T}^{4}$.Assume ${T}^{4}$ as the difference of absolute temperature and temperature at the lower of wall. Now expand the ${T}^{4}$ upto two term by Taylor series we got,

7 $${T}^{4}\approx 4{T}_{2}^{4}.T-3{T}_{2}^{4},$$

Use relation (6) and (7) in Eq. (4), then we obtain,

8 $$({\rho Cp)}_{hbf}\left(u\frac{\partial T}{\partial x}+v\frac{\partial T}{\partial y}\right)={K}_{bsf}\left(\frac{{K}_{hbf}}{{K}_{bsf}}+\frac{16{\sigma }^{*}{T}_{2}^{3}}{3{k}^{*}{K}_{bsf}}\right)\frac{{\partial }^{2}u}{\partial {y}^{2}}+\tau ({\rho Cp)}_{hbf}\left(\frac{{D}_{B}{\chi }_{c}}{a}\frac{\partial T}{\partial y}+\frac{{D}_{T}}{{T}_{2}} {\left(\frac{\partial T}{\partial y}\right)}^{2}\right)+{\mu }_{hbf}{u}^{2},$$

### Dimensionless equations with similarity solution

To dimensionless the above Eq. (1)–(8) with the help of wall boundaries (5) after eliminate the pressure term $P$ there are modified some similarity transformations which are,

9 $$u=-\frac{x}{{a}^{2}}.\frac{{\mu }_{f}}{{\rho }_{f}}{f}^{{\prime}\left(\eta \right)}, v=\frac{1}{a}.\frac{{\mu }_{f}}{{\rho }_{f}}f\left(\eta \right), \theta =\frac{T-{T}_{2}}{{T}_{2}-{T}_{1}}, \eta =\frac{y}{a}.$$

After simplify and applying the similarity transformations, we got,

10 $$\frac{{\mu }_{hbf}/{\mu }_{bsf}}{{\rho }_{hbf}/{\rho }_{bsf}}\left(1+\frac{1}{{\beta }_{c}}\right) {F}^{iv}+Re\left({F}^{\prime}{F}^{{\prime}{\prime}}-F{F}^{{\prime}{\prime}{\prime}}\right)+\frac{M{F}^{{\prime}{\prime}}}{{\rho }_{hbf}/{\rho }_{bsf}}=0,$$

11 $$\frac{1}{Pr.{\left(\rho CP\right)}_{hbf}/{\left(\rho CP\right)}_{bsf}}\left(\frac{{K}_{hbf}}{{K}_{bsf}}+\frac{4}{3}{\Gamma }_{ad}\right){\theta }^{{\prime}{\prime}}+{N}_{b}{\theta }^{\prime}+{N}_{t}{\theta }^{{\prime}2}+\frac{{\mu }_{hbf}/{\mu }_{bsf}}{{(\rho CP)}_{hbf}/({\rho CP)}_{bsf}}{E}_{c}{F}^{{\prime}2}-F{\theta }^{\prime}=0,$$

With boundaries of stretching and shrinking channel,

12 $$\begin{aligned} & F^{\prime}=\mp 1, F=0, \theta =1 \,at \,upper \,plate \left(\eta =-3\right),\\ &F^{\prime}=\mp 1, F=0, \theta =0 \,at \,lower \,plate \left(\eta =3\right).\end{aligned}$$

where $Re=\frac{{\rho }_{bsf} {a}^{2}{U}_{w}}{{\mu }_{bsf}}$ is permeable Reynolds number, $M=\frac{{\sigma }_{s}{B}_{0}^{2}{a}^{2}}{{\mu }_{bsf}}$ is magnetic parameter, $Pr=\frac{{\left(\mu \right)}_{bsf}{\left(CP\right)}_{bsf}}{{\mu }_{bsf}}$ is prendtl number,${\Gamma }_{ad}=\frac{{\sigma }_{c}{T}_{2}^{3}}{3{K}^{*}{K}_{bsf}}$ is the thermal radiation parameter,${N}_{b}=\frac{{\rho }_{bsf}\tau {D}_{B}{\upchi }_{{\text{c}}}}{{\mu }_{bsf}}$ is Brownian motion parameter, ${N}_{t}=\frac{{\rho }_{bsf}\tau {D}_{T}\mathrm{\Delta T}}{{\mu }_{bsf}{T}_{2}}$ is Thermophoresis motion parameter and ${E}_{c}={\left(\frac{x{\mu }_{bsf}}{{a\rho }_{bsf}}\right)}^{2}\frac{1}{{\left(CP\right)}_{bsf}}$ denoted as Eckert number.

## Applications

### Numerical solution of problem

The finite element method (NFEM) is a robust numerical approach utilized to tackle intricate engineering and mathematical problems. It finds extensive applications across diverse fields such as structural analysis, fluid dynamics, heat transfer, and electromagnetic. In the finite element method, the subsequent stage entails estimating the behavior of the unknown function within each element by employing interpolation functions. These functions, commonly referred to as shape functions, are formulated based on the element's properties and serve to represent the unknown function's behavior within the element. By combining these shape functions with the established equations that govern the problem, a system of equations is constructed for each element.

Assume that,

13 $${F}^{\prime}=G,$$

Then the Eqs. (12) and (13) becomes,

14 $${\lambda }_{1}{\lambda }_{2}^{-1}\left(1+{\beta }^{-1}\right){G}^{{\prime}{\prime}{\prime}}+Re\left(G{F}^{\prime}-FG^{{\prime}{\prime}}\right)+M{G}^{\prime}{\lambda }_{2}^{-1}=0,$$

15 $$\frac{{\lambda }_{3}^{-1}}{Pr }\left({\lambda }_{4}+\frac{4}{3}{\Gamma }_{ad}\right){H}^{\mathrm{^{\prime}}\mathrm{^{\prime}}}{+}^{\mathrm{^{\prime}}}\left(Nb+Nt{H}^{\mathrm{^{\prime}}}-F\right){H}^{\mathrm{^{\prime}}}+{\lambda }_{1}{\lambda }_{3}^{-1}Ec{G}^{2}=0.$$

Subjected to boundaries,

16 $$f\left(-3\right)=0;f\left(3\right)=0;G\left(-3\right)=\mp 1;G\left(3\right)=\pm 1;H\left(-3\right)=1;H\left(3\right)=0.$$

Now apply the weighted residual method to discretize the whole domain $\left({\eta }_{e},{\eta }_{e+1}\right).$

17 $$\underset{{\eta }_{e}}{\overset{{\eta }_{e+1}}{\int }}{R}_{1}\left({F}^{\prime}-G\right)=0,$$

18 $$\underset{{\eta }_{e}}{\overset{{\eta }_{e+1}}{\int }}{R}_{2}\left({\lambda }_{1}{\lambda }_{2}^{-1}\left(1+{\beta }^{-1}\right){G}^{\mathrm{^{\prime}}\mathrm{^{\prime}}\mathrm{^{\prime}}}+Re\left(G{F}^{\mathrm{^{\prime}}}-FG\mathrm{^{\prime}}\mathrm{^{\prime}}\right)+M{G}^{\mathrm{^{\prime}}}{\lambda }_{2}^{-1}\right)=0,$$

19 $$\underset{{\eta }_{e}}{\overset{{\eta }_{e+1}}{\int }}{R}_{3}\left(\frac{{\lambda }_{3}^{-1}}{Pr }\left({\lambda }_{4}+\frac{4}{3}{\Gamma }_{ad}\right){H}^{{\prime}{\prime}}{+}^{\prime}\left(Nb+Nt{H}^{\prime}-F\right){H}^{\prime}+{\lambda }_{1}{\lambda }_{3}^{-1}Ec{G}^{2}\right)=0.$$

where ${R}_{1},{R}_{2},{R}_{3}={\psi }_{ij}, \forall i, j=\mathrm{1,2},3.$ Then the quadratic shape function $\psi$ and approximation solution for non-linearity defined as,

$${\psi }_{1}^{e}=\frac{\left({\eta }_{e+1}-\eta \right)\left({\eta }_{e+1}+{\eta }_{e}-2\eta \right)}{{\left({\eta }_{e+1}-{\eta }_{e}\right)}^{2}}, {\psi }_{2}^{e}=\frac{4\left(\eta -{\eta }_{e}\right)\left({\eta }_{e+1}-\eta \right)}{{\left({\eta }_{e+1}-{\eta }_{e}\right)}^{2}},$$

20 $${\psi }_{3}^{e}=\frac{\left(\eta -{\eta }_{e}\right)\left({\eta }_{e+1}+{\eta }_{e}-2\eta \right)}{{\left({\eta }_{e+1}-{\eta }_{e}\right)}^{2}},$$

21 $$F=\sum_{j=1}^{3}{F}_{j}{\psi }_{j}, G=\sum_{j=1}^{3}{G}_{j}{\psi }_{j}.$$

where, $${\eta }_{e}\le \eta \le {\eta }_{e+1}.$$

After simplify the above equation we get the finite element modal for the solution.

22 $$\left[{K}^{e}\right]\left[{S}^{e}\right]=\left[{B}^{e}\right],$$

where ${[K}^{e}]$ denotes the stiffness matrix, ${[S}^{e}]$ denoted the vector nodal points for unknown values and ${[B}^{e}]$ denotes the right hand side of the above equations shows below.

23 $${[K}^{e}]=\left[\begin{array}{ccc}\left[{K}^{11}\right]& \left[{K}^{12}\right]& \left[{K}^{13}\right]\\ \left[{K}^{21}\right]& \left[{K}^{22}\right]& \left[{K}^{23}\right]\\ \left[{K}^{31}\right]& \left[{K}^{32}\right]& \left[{K}^{33}\right]\end{array}\right], \left[{S}^{e}\right]=\left[\begin{array}{c}\{F\}\\ \{G\}\\ \{H\}\end{array}\right], \left[{B}^{e}\right]=\left[\begin{array}{c}\{{b}^{1}\}\\ \{{b}^{2}\}\\ \{{b}^{3}\}\end{array}\right],$$

where each $\left[K\right]$ is order of $3\times 3$ and each $\left[b\right]$ is matrix of order $3\times 1$, defined after simplification,

24 $$\begin{aligned} & {K}_{ij}^{11}=\underset{{\eta }_{e}}{\overset{{\eta }_{e+1}}{\int }}{\psi }_{i}{\psi }_{j}^{\prime}d\eta , {K}_{ij}^{12}=\underset{{\eta }_{e}}{\overset{{\eta }_{e+1}}{\int }}{\psi }_{i}{\psi }_{j}d\eta , {K}_{ij}^{13}=0,{K}_{ij}^{21}=0, &\underset{{\eta }_{e}}{\overset{{\eta }_{e+1}}{\int }}\left(-{\lambda }_{1}{\lambda }_{2}^{-1}\left(1+{\beta }^{-1}\right){\psi }_{i}{\psi }_{j}^{{\prime}{\prime}}-Re{\psi }_{i}\overline{F}{\psi }_{j}^{{\prime}{\prime}}+Re{\psi }_{i}\stackrel{-}{F^{\prime}}{\psi }_{j}^{\prime}+M{\lambda }_{2}^{-1}{\psi }_{i}\stackrel{-}{F^{\prime}}{\psi }_{j}\right)d\eta =0,\\& {K}^{23}=0, {K}^{31}=0,\\ & {K}^{32}={\lambda }_{1}{\lambda }_{3}^{-1}Ec \underset{{\eta }_{e}}{\overset{{\eta }_{e+1}}{\int }}{\psi }_{i}\overline{G}{\psi }_{j}d\eta ,\\ & {K}^{33}=\underset{{\eta }_{e}}{\overset{{\eta }_{e+1}}{\int }}\left(-\frac{{\lambda }_{3}^{-1}}{Pr}\left({\lambda }_{4}+\frac{4}{3}{\Gamma }_{ad}\right){\psi }_{i}^{\prime}{\psi }_{j}^{\prime}+(Nb+Nt\overline{{H }^{\prime}}-\overline{F }){\psi }_{i}{\psi }_{j}^{\prime}\right)d\eta =0,\\ &{b}^{1}=0, {b}^{2}=-{\lambda }_{1}{\lambda }_{2}^{-1}\left(1+{\beta }^{-1}\right){\left. {{\text{R}}}_{2}\mathrm{G^{\prime}}\mathrm{^{\prime}}\right|}_{{\eta }_{e}}^{{\eta }_{e+1}}, {b}^{2}=-\frac{{\lambda }_{3}^{-1}}{Pr}\left({\lambda }_{4}+\frac{4}{3}{\Gamma }_{ad}\right){\left. {{\text{R}}}_{3}\mathrm{H^{\prime}}\right|}_{{\eta }_{e}}^{{\eta }_{e+1}}. \end{aligned}$$

Here,

25 $$\stackrel{-}{F^{\prime}}=\sum_{j=1}^{3}\overline{{F }_{j}}{\psi }_{j},\stackrel{-}{G^{\prime}}=\sum_{j=1}^{3}\overline{{G }_{j}}{\psi }_{j}.$$

The finite element technique employs a unique property where, instead of seeking an approximate solution across the entire region, the region is partitioned into smaller elements. Subsequently, each element is approximated individually. By discerning the characteristics of each element and combining their components, we strive to attain a preliminary understanding of a solution applied to the entire body. In our present scenario, we divided the domain's length into $(601)$ points of total length $(\Delta \eta =6)$, resulting in a total of $(1203)$ quadratic shape elements. Through the assembly process, we obtained an order matrix of size $(3609\times 3609)$ and $(3603)$ linear equations for each of the three functions. These equations were then solved using the Gaussian elimination method, utilizing python programming language. This process is repeated iteratively until reaching a convergence control tolerance of $(1e-6)$.

## Results and discussion

The finite element method was used to handle the numerical results of nonlinear differential Eqs. (10) and (11) with the appropriate boundary conditions (12). These highly nonlinear coupled ODEs are solved with the help of finite element technique after discretization of the elements. The performance of the variables concerning velocity and temperature distributions was examined. We discussed the values for the flowing parameters such as the Casson fluid parameter ${\beta }_{c}$, permeable Reynolds number $(Re)$, magnetic field parameter $(M)$, the Brownian motion parameter $({N}_{b})$, Thermophoresis motion parameter $({N}_{t})$, the volume of fraction of both nanoparticles $({\phi }_{p1}, {\phi }_{p2})$ and Prandtl number $(Pr)$. All of these values are kept constant except the variable one for each computation, which is significant in graphs and tables. These parameters’ effects on physical quantities like $\left|{F}^{{\prime}{\prime}}\left(-3\right)\right|$ and $|\theta ^{\prime}(-3)|$ which will be the main focus of the study. The velocity $F^{\prime}(\eta )$ and temperature $\theta (\eta )$ profiles are represented in a discussion by prevalence. Thermophysical properties of Casson fluid and nanoparticles $(MWCONTs, A{l}_{2}{O}_{3})$ are describes in^32^. To corroborate our findings, we utilized pertinent research articles from the past to illustrate the data (see in Table 3). An impressive resemblance has been established, confirming the reliability of the finite element method implemented with the Python programming language. The observed numerical improvement is reflected in the significant contrast.

**Table 3:** Comparison results with *Raza *et al*.*^27^ for ${\beta }_{c}$ after fixing the values of $M=2, Pr=6.2, {\Gamma }_{d}=3, Ec=0.1, {\phi }_{p1}={\phi }_{p2}=0.01, {N}_{b}={N}_{t}=0.1.$

${\beta }_{c}$	*Raza *et al*.*^27^				Present results			
	$Re>0$		$Re<0$		$Re>0$		$Re<0$	
	Shear stress	Heat transfer	Shear stress	Heat transfer	Shear stress	Heat transfer	Shear stress	Heat transfer
0.2	2.7869	0.1377	2.7544	0.1376387	2.7861782	0.1374302	2.7861782	0.1374302
0.4	2.6234	0.1370	2.5982	0.1371574	2.6234571	0.1373941	2.6234571	0.1373941
0.6	2.4936	0.1365	2.4911	0.1366587	2.49354451	0.1364421	2.49354451	0.1364421
0.8	2.3878	0.1361	2.4135	0.1361482	2.3876773	0.1363147	2.3876773	0.1363147

When observing the results for $F^{{\prime}{\prime}} (-3)$ and $F^{{\prime}{\prime}}(3)$ with varying magnetic parameters in both stretching and shrinking cases, it becomes evident that there is a direct correlation (see in Table 4). However, an opposite trend emerges when the magnetic parameter's value is increased. Specifically, for the stretching case, increasing the magnetic parameter leads to an increase in heat transfer rate between both plates of the channel. Conversely, in the shrinking case, an increase in the magnetic parameter causes a decrease in the heat transfer rate between the plates.

**Table 4:** Variation results for $M$ by fixing ${\beta }_{c}=0.5,Re=4, Pr=6.2, {\Gamma }_{d}=3, Ec=0.1, {\phi }_{p1}={\phi }_{p2}=0.01, {N}_{b}={N}_{t}=0.1.$

$M$	Stretching case				Shrinking case			
	$|F^{{\prime}{\prime}}(-3)|$	$|F^{{\prime}{\prime}}(3)|$	$|\theta^{\prime}(-3)|$	$|\theta ^{\prime}(3)|$	$|F^{{\prime}{\prime}}(-3)|$	$|F^{{\prime}{\prime}}(3)|$	$|\theta ^{\prime}(-3)|$	$|\theta^{\prime}(3)|$
2	1.0271356	1.0271655	0.0216521	0.021652133	1.2875921	1.2875908	0.194474	0.194472
3	1.2287963	1.2287916	0.0344199	0.034419966	1.4892527	1.4892569	0.173439	0.173447
4	1.4015626	1.4015611	0.0445366	0.044536633	1.6620190	1.6620164	0.158618	0.158682
5	1.5454346	1.5454546	0.0512892	0.051289287	1.8058909	1.8058993	0.149374	0.149391
6	1.6604121	1.6604103	0.0542537	0.054253784	1.9208685	1.9208656	0.145316	0.145346

The effect of the Casson parameter and permeable Reynolds number for both cases is presented in Tables 5 and 6. It is observed that in the stretching case, increasing the values of the Casson parameter leads to a rise in both shear stress and heat transfer rate for both plates of the channel. However, the results contradict this trend in the shrinking case (refer to Table 5). Similarly, increasing the values of the Reynolds number causes a decrease in shear stress for the stretching case but an increase for the shrinking case. There is a direct relationship between the heat transfer rate and Reynolds number for both plates in both the stretching and shrinking cases. By increasing the values of the Reynolds number, the heat transfer rate is enhanced for both cases.

**Table 5:** Variation results for ${\beta }_{c}$ by fixing $M=0,Re=4, Pr=6.2, {\Gamma }_{d}=3, Ec=0.1, {\phi }_{p1}={\phi }_{p2}=0.01, {N}_{b}={N}_{t}=0.1.$

	Hybrid nanofluids $({\text{MWCONTs}}-{{\text{Al}}}_{2}{{\text{O}}}_{3})/{\text{blood}}$							
${\beta }_{c}$	Stretching case				Shrinking case			
	$|F^{{\prime}{\prime}}(-3)|$	$|F^{{\prime}{\prime}}(3)|$	$|\theta ^{\prime}(-3)|$	$|\theta ^{\prime}(3)|$	$|F^{{\prime}{\prime}}(-3)|$	$|F^{{\prime}{\prime}}(3)|$	$|\theta ^{\prime}(-3)|$	$|\theta ^{\prime}(3)|$
0.2	0.631231	0.631241	0.0346124	0.034612	2.7867	2.78682	0.13743	0.13702
0.4	2.720869	2.720896	0.0386477	0.038647	2.6235	2.62371	0.13739	0.13741
0.6	3.640204	3.640225	0.0409642	0.040964	2.4934	2.49351	0.13644	0.13621
0.8	4.016200	4.016285	0.0423875	0.042387	2.3877	2.38773	0.13631	0.13647
1.0	4.122862	4.122843	0.0433065	0.043306	2.2184	2.218545	0.136259	0.13679

**Table 6:** Variation results for $Re$ by fixing $M=0,{\beta }_{c}=0.5, Pr=6.2, {\Gamma }_{d}=3, Ec=0.1, {\phi }_{p1}={\phi }_{p2}=0.01, {N}_{b}={N}_{t}=0.1.$

$Re$	Stretching case				Shrinking case			
	$|F^{{\prime}{\prime}}(-3)|$	$|F^{{\prime}{\prime}}(3)|$	$|\theta ^{\prime}(-3)|$	$|\theta ^{\prime}(3)|$	$|F^{{\prime}{\prime}}(-3)|$	$|F^{{\prime}{\prime}}(3)|$	$|\theta ^{\prime}(-3)|$	$|\theta ^{\prime}(3)|$
3	0.7999043	0.7999014	0.015050	0.015040	2.056228	2.056226	0.276201	0.276231
4	0.5977914	0.5977363	0.028058	0.028058	2.272889	2.272892	0.318945	0.318945
5	0.3977567	0.3977529	0.034612	0.034672	2.491629	2.491697	0.349006	0.349079
6	0.1998005	0.1998011	0.036513	0.036583	2.712448	2.712484	0.365575	0.365586
7	0.0039226	0.0039277	0.039582	0.039522	2.935345	2.935321	0.388701	0.388788

The outcomes of heat transfer rate for the stretching and shrinking scenarios, obtained by varying the values of the remaining physical parameter ${N}_{b},{N}_{t},{E}_{c},{\Gamma }_{d},{\phi }_{p1},{\phi }_{p2}$, are depicted in Table 6. As the Brownian motion parameter is augmented, it results in an opposite correlation for both stretching and shrinking cases. The heat transfer rate diminishes with the escalation of the Brownian motion parameter in the shrinking case, whereas it escalates with the augmentation of the Brownian motion parameter in the stretching case. The Thermophoresis mobility parameter yields contrary outcomes compared to the heat transfer rate and Brownian motion impact for both stretching and shrinking scenarios. When the Thermophoresis mobility parameter is augmented, the heat transfer rate diminishes during stretching, while it escalates by elevating the value of the said parameter during shrinking.

Thermal radiation and Eckert number are the reasons behind the improved heat transfer rate. When you raise the values of the thermal radiation parameter and the Eckert number, the heat transfer rate increases for both the channel's surfaces in both stretching and shrinking scenarios. The Prandtl number, which represents the ratio of momentum diffusivity to thermal diffusivity in a hybrid type nanofluids, plays a significant role in determining the heat transfer rate. When studying the heat transfer phenomenon in both stretching and shrinking scenarios, it has been found that higher values of the Prandtl number lead to a noticeable decrease in the heat transfer rate on both surfaces of the channel. The heat transfer rate of hybrid Casson nanofluids is significantly influenced by the volume concentration of nanoparticles. Observations reveal that when the percentages of one variable are varied while keeping the other fixed, the heat transfer rate shows a slight increase in both stretching and shrinking cases. This suggests that enhancing the volume concentration might lead to improved thermal performance in the flow.

**Figure 2:**
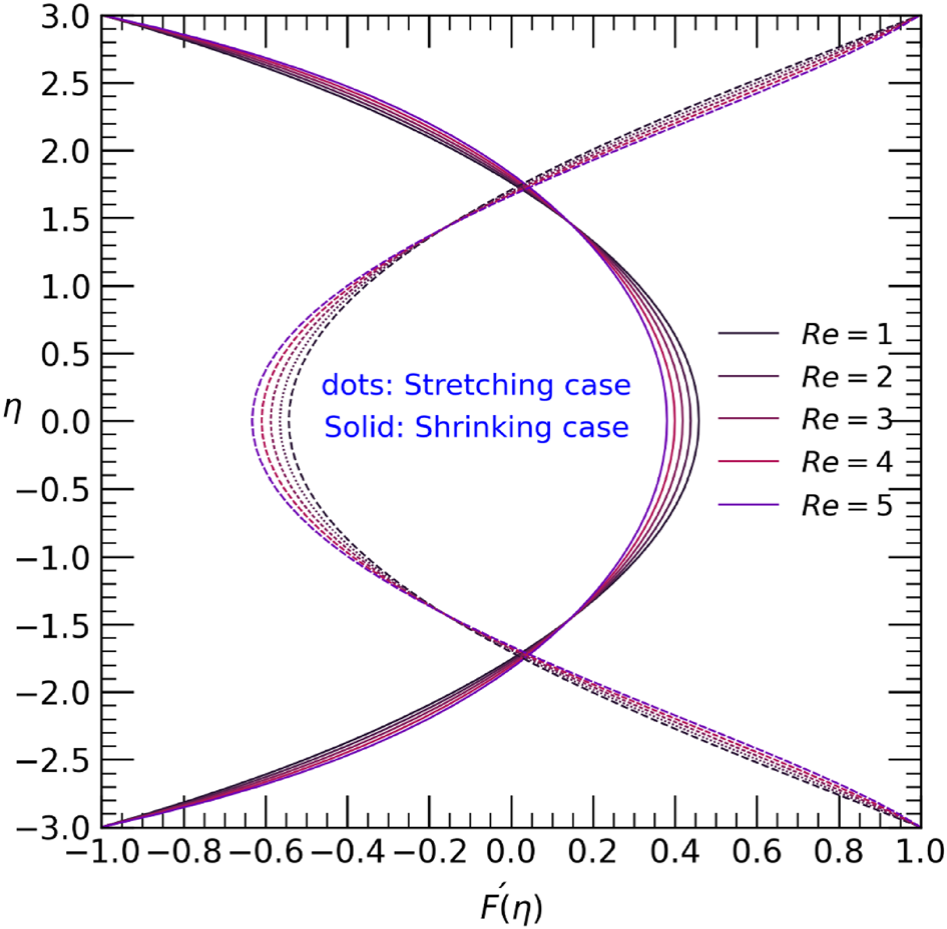
Velocity profile with effect to Reynolds number $Re$.

**Figure 3:**
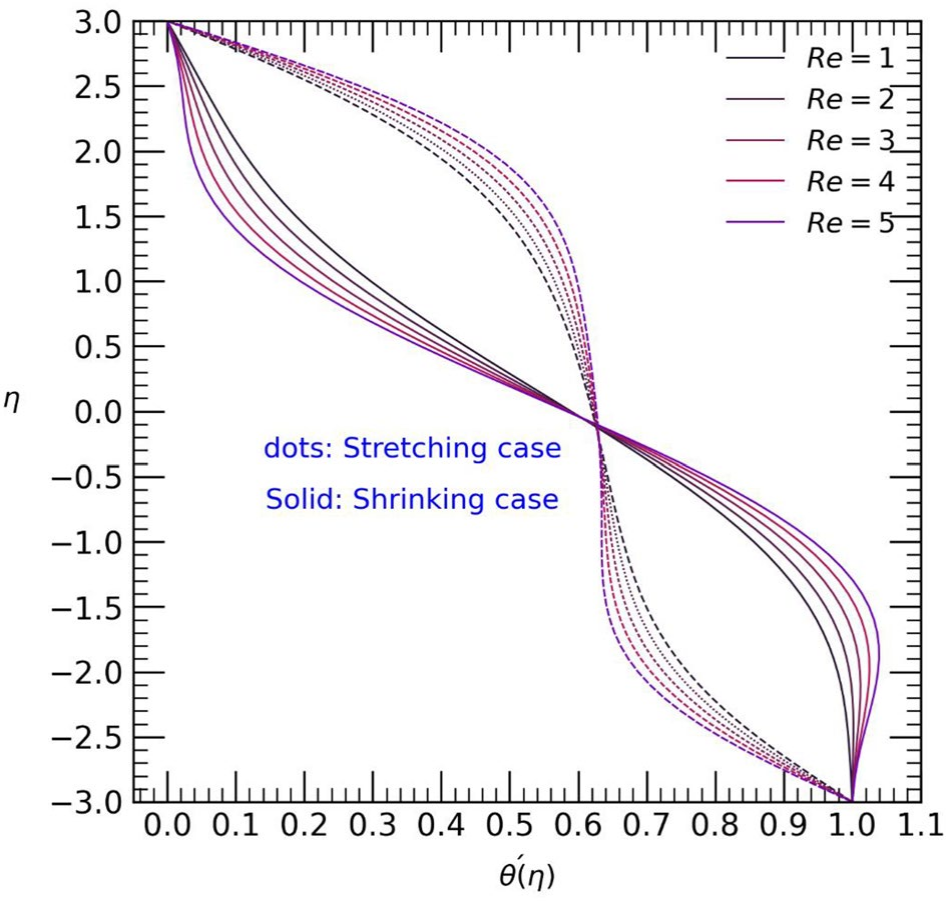
Temperature profile with effect to Reynolds number $Re$.

**Figure 4:**
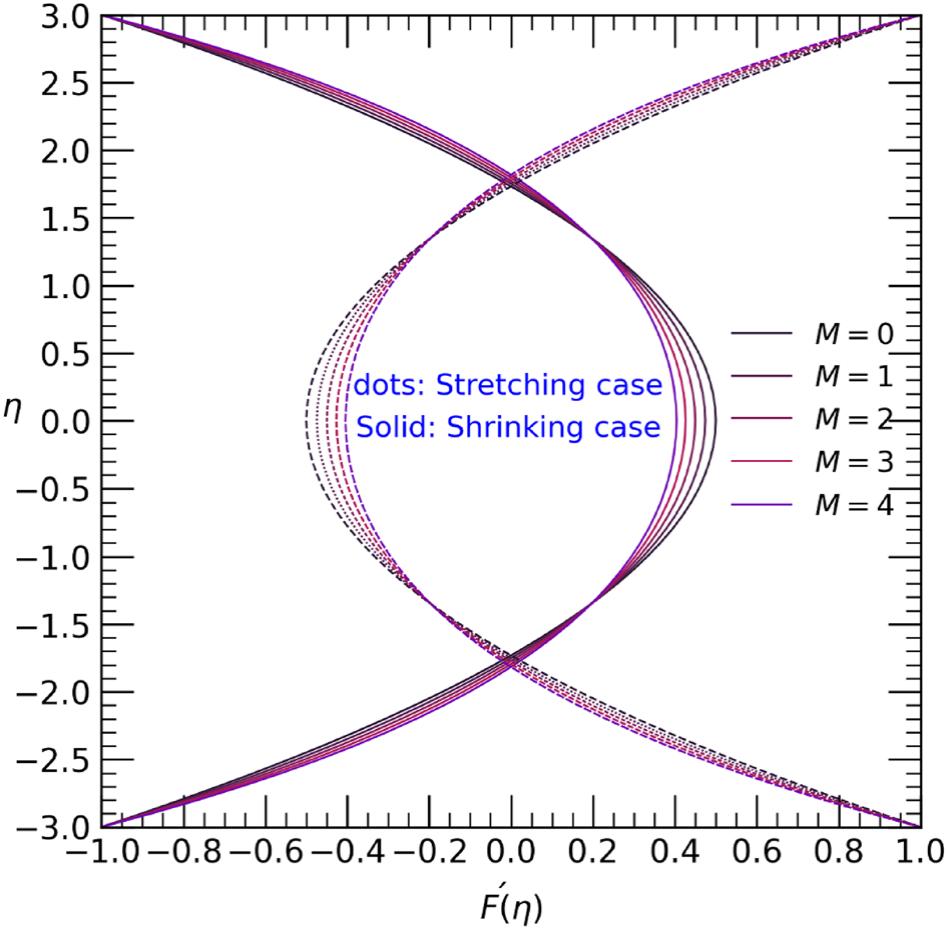
Velocity profile with effect to magnetic parameter $M$.

**Figure 5:**
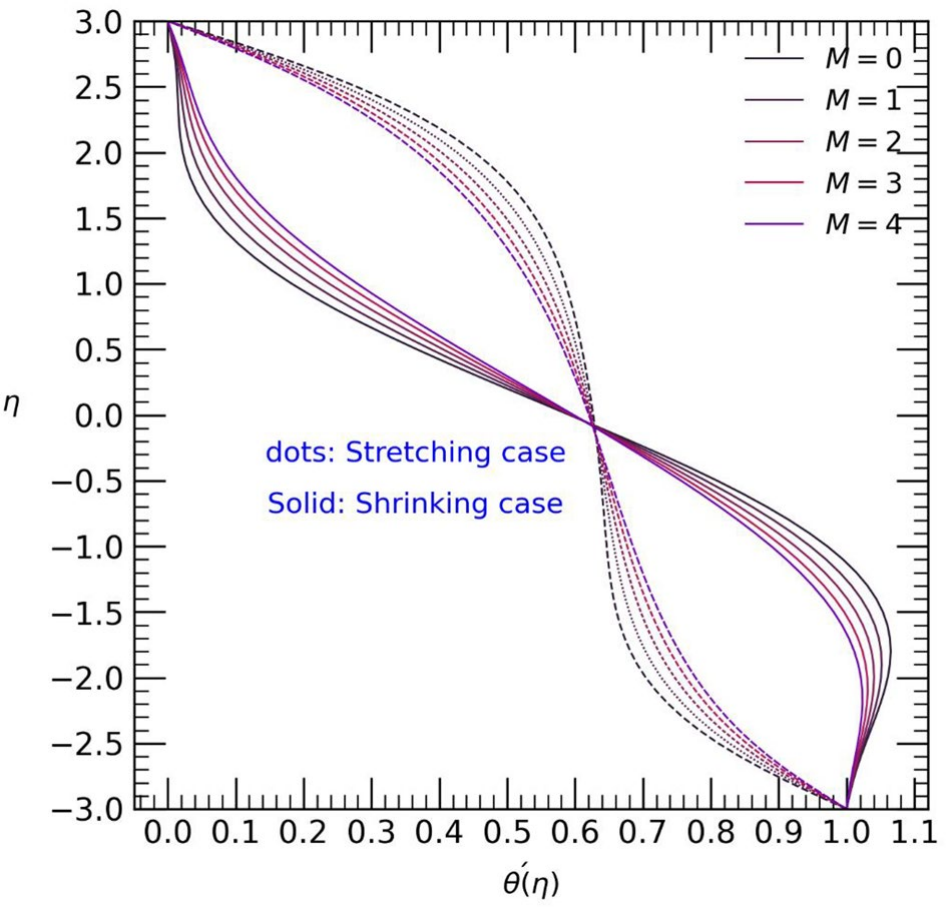
Temperature profile with effect to magnetic parameter $M$.

**Figure 6:**
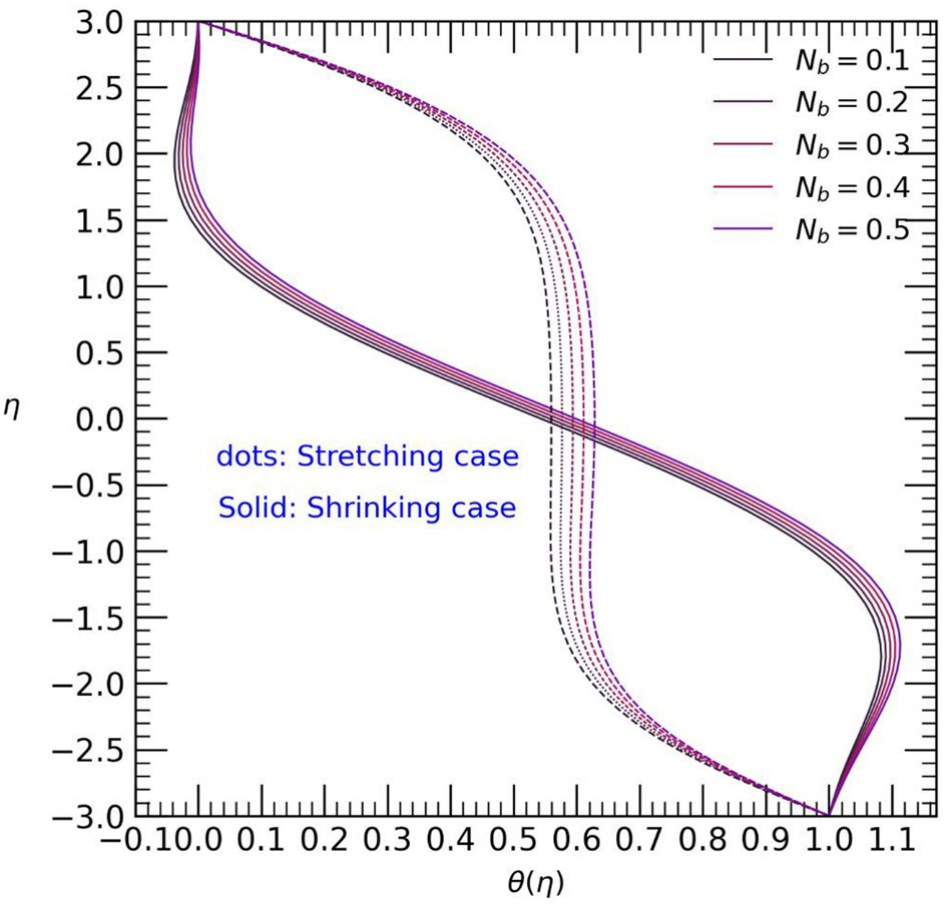
Temperature profile with effect to Brownian motion ${N}_{b}$.

**Figure 7:**
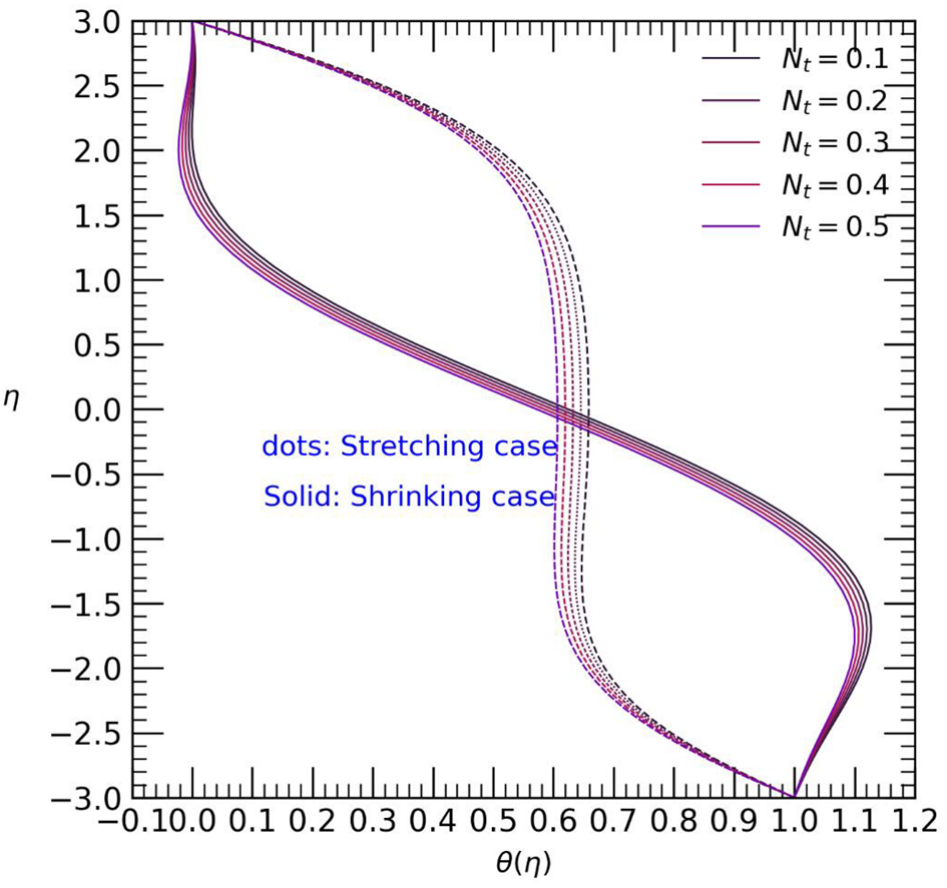
Temperature profile with effect to Thermophoresis motion ${N}_{t}$.

**Figure 8:**
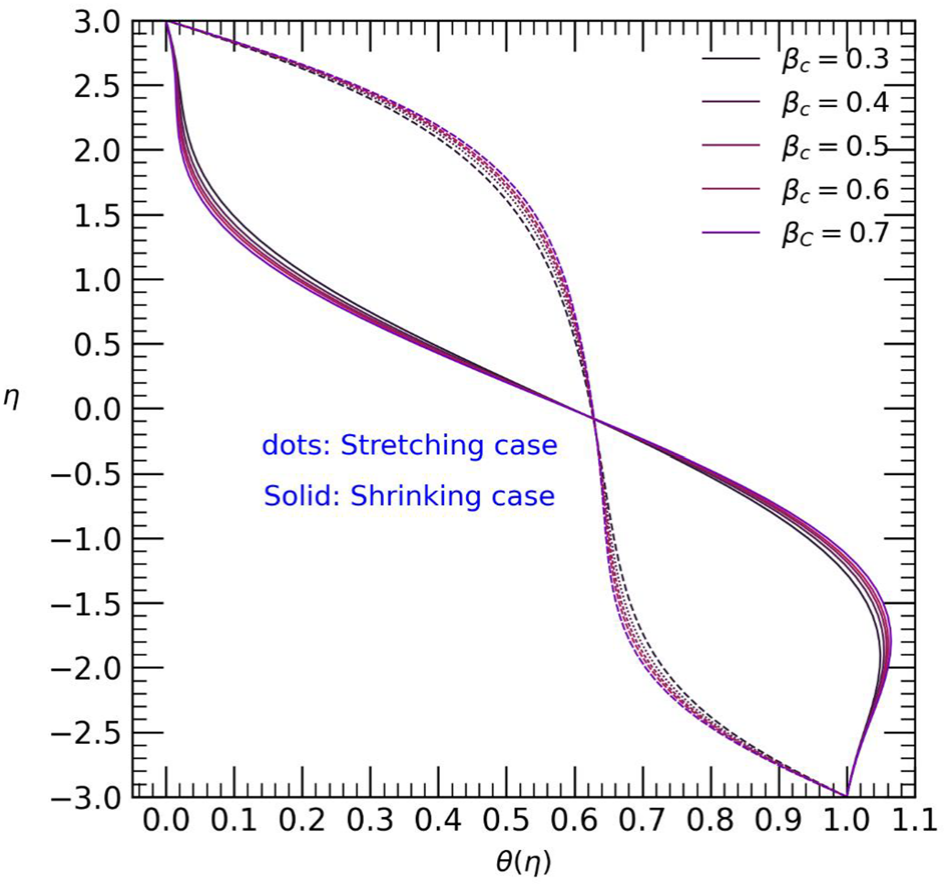
Temperature profile with effect to Casson parameter ${\beta }_{c}$.

**Figure 9:**
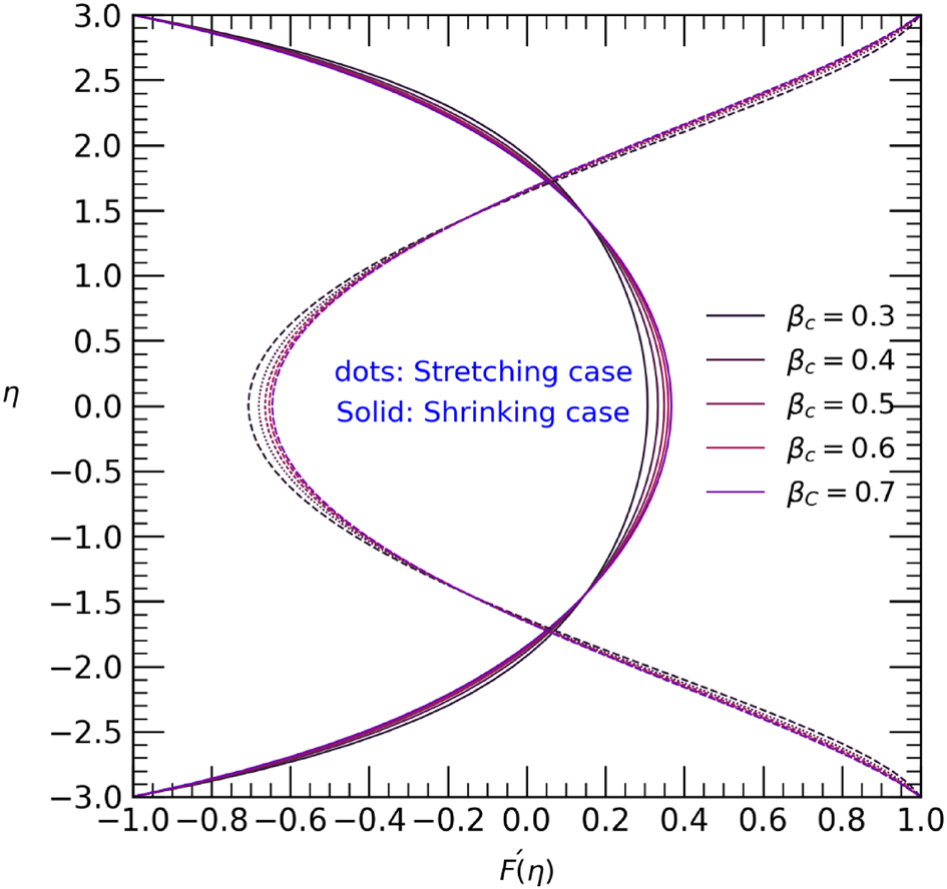
Velocity profile with effect to Casson parameter ${\beta }_{c}$.

**Figure 10:**
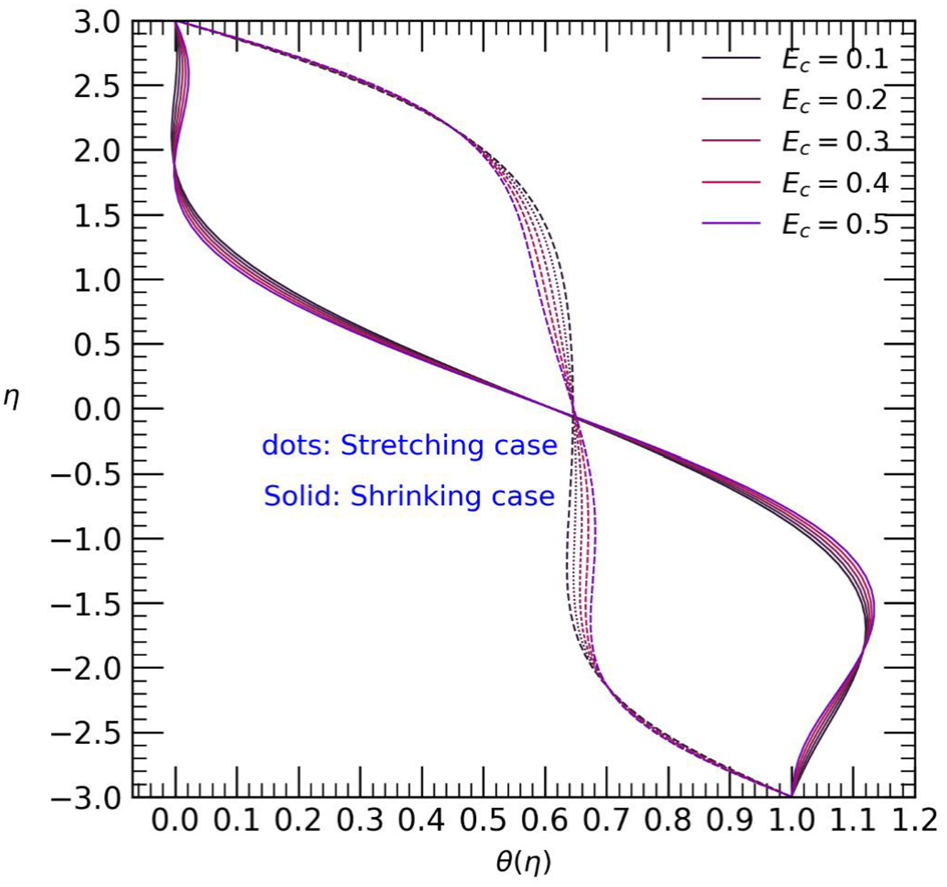
Temperature profile with effect to Eckert number ${E}_{c}$.

**Figure 11:**
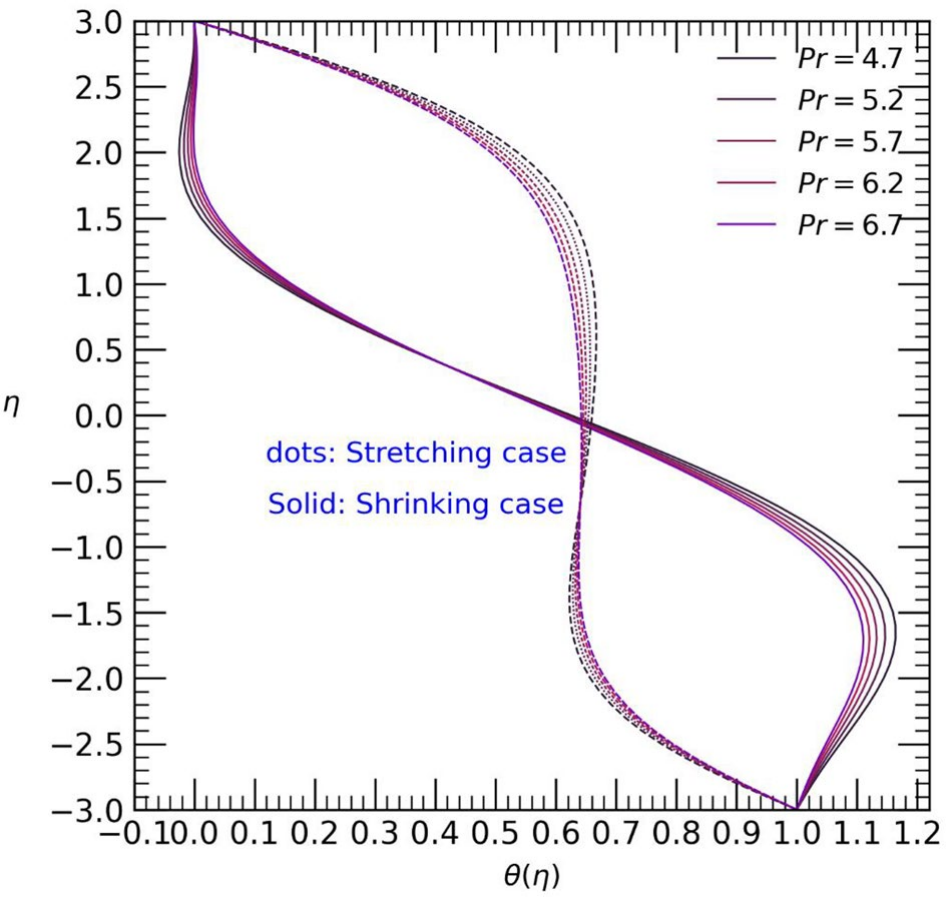
Temperature profile with effect to Prandtl number $Pr$.

**Figure 12:**
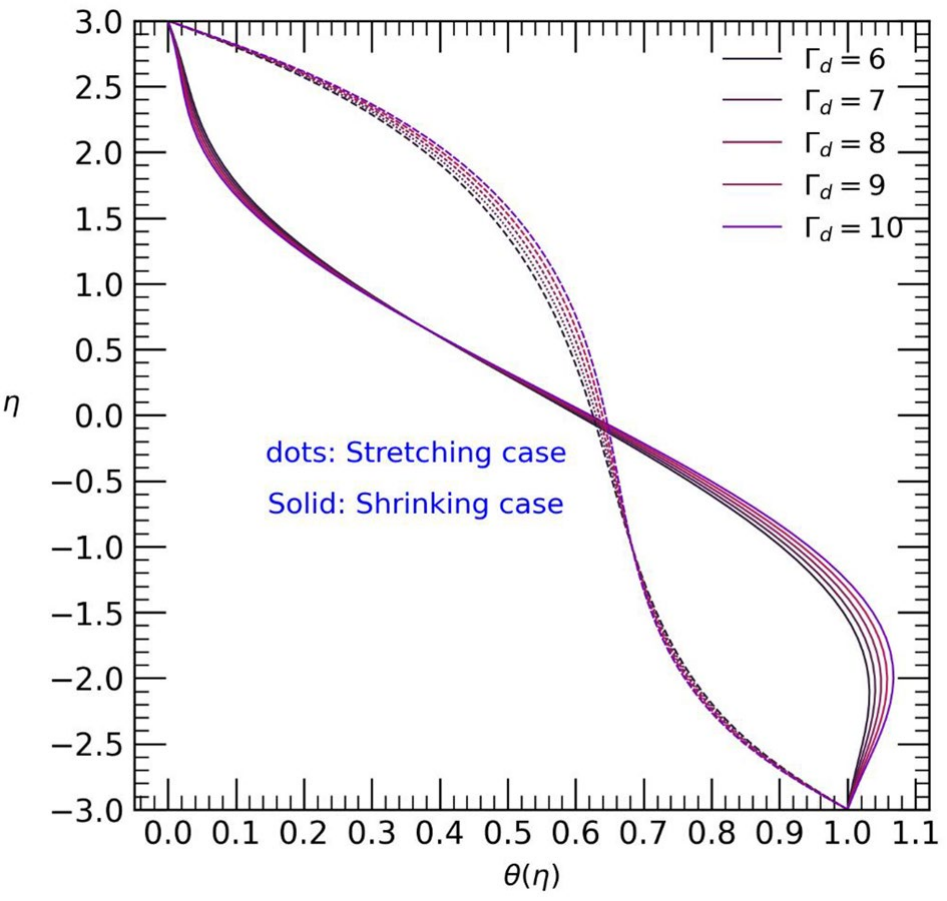
. Temperature profile with effect to thermal radiation parameter ${\Gamma }_{d}$.

Different variation of the physical parameters varying one of them after fix constant values of others the velocity $F^{\prime}(\eta )$ and temperature $\theta (\eta )$ are shown in Figs. 2, 3, 4, 5, 6, 7, 8, 9, 10, 11 and 12. Figure 2 illustrates the impact of the Reynolds number $Re$ on the velocity profile $F{\prime}(\eta )$ when the wall undergoes stretching and contracting. It can be observed that as the Reynolds number $Re$ increases, the velocity decreases in the center of the channel but increases near the bottom and upper walls in the case of wall contraction. Conversely, in the stretching case, an opposite trend is observed, where the velocity near the bottom and upper walls decreases while increasing in the center of the channel as the Reynolds number $Re$ rises.

Figure 3 illustrates the correlation between the temperature profile $\theta (\eta )$ and the permeable Reynolds number $Re$. It is evident that the hybrid Casson nanofluids exhibit opposite behaviors in the stretching and shrinking cases near the upper and lower walls of the channel. In the stretching case, the temperature gradually increases from the upper wall towards the center of the channel, but decreases near the lower wall. On the other hand, in the shrinking case, the opposite trend is observed.

The influence of the magnetic field parameter $M$ on both the temperature $\theta (\eta )$ and velocity profiles $F{\prime}(\eta )$ is depicted in Fig. 4 and 5. The application of a magnetic field leads to a decrease in velocity towards the center of the channel in the shrinking case, while an opposite effect is observed in the stretching case (refer to Fig. 4). Greater variation of velocity into center of channel due to some external forces, may be some frictional forces and Lorent’z forces effects on the fluid flow. Similarly, in the stretching case, the temperature near the upper wall decreases but increases from the center to the lower wall. However, in the shrinking case, with an increase in the magnetic field parameter $M$, the graphical results for temperature show the opposite trend (see Fig. 5).

The increase in Brownian motion ${N}_{b}$ and Thermophoresis motion parameter ${N}_{t}$ for positive values leads to a steady rise in temperature from both walls towards the center of the channel (refer to Figs. 6 and 7). In the stretching case, the rise in Brownian motion ${N}_{b}$ results in an increase in temperature towards the center of the channel, while the opposite effect is observed in the shrinking case. Moreover, an increase in the values of the thermophoresis motion parameter ${N}_{t}$ gradually elevates the temperature towards the center of the channel for both stretching and shrinking cases (see Fig. 7). This temperature rise may be attributed to the presence of colloidal nanoparticles of both types.

The Casson parameter significantly influences the flow of non-Newtonian fluids in hybrid nanofluids and may find practical applications in scenarios like blood flow in veins. Increasing the Casson parameter values results in an anti-symmetrical temperature distribution near the walls of the channel. Specifically, in the stretching case (Fig. 8), the temperature rises from the upper walls towards the center of the channel, while it decreases from the center to the lower wall. Conversely, in the shrinking case, the temperature follows the opposite trend. In Fig. 9, for both stretching and shrinking cases, an increase in the Casson parameter values leads to an enhanced velocity in a small area at the center of the channel, but the changes near both walls of the channel are relatively minor. Consequently, as the Casson parameter values increase, the graphical results of the velocity profile show symmetric decreases near both the upper and lower walls in each case.

Figure 10 displays the temperature profile $\theta (\eta )$ obtained by varying the non-negative values of the Eckert number ${E}_{c}$. The graph reveals that the temperature symmetrically increases near the boundaries of the channel for both cases. However, small changes are observed from both walls towards the center. Specifically, the temperature decreases near the upper wall but increases towards the lower wall from the center to the boundaries of the bottom wall as the Eckert number ${E}_{c}$ increases.

**Figure 13:**
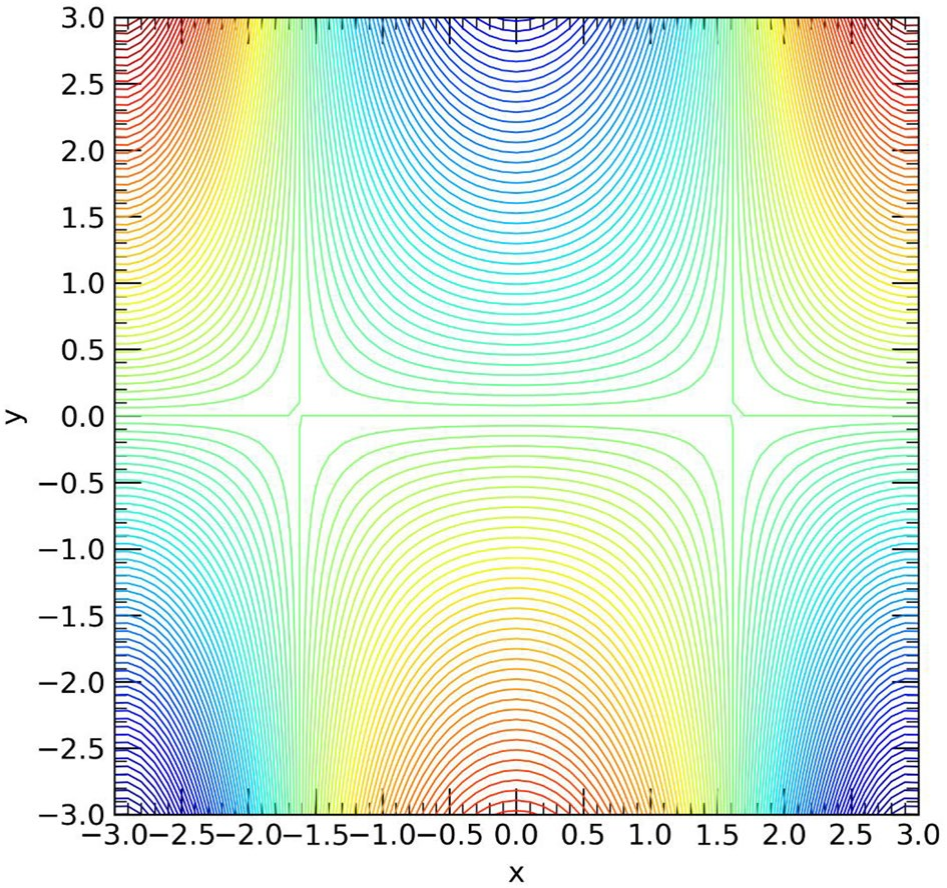
Contour plot of velocity profile for $Re=4, M=1, {\phi }_{p1}={\phi }_{p2}=0.1, {\beta }_{c}=0.5$.

Figure 11 and 12 illustrates the impact of the Prandtl number $Pr$ and thermal radiation ${\Gamma }_{d}$ on the temperature profile $\theta (\eta )$. Both parameters show a slight coincidence with each other for each case. As the values of the Prandtl number $pr$ increase, the temperature decreases from the upper wall towards the center of the channel, but it promptly increases from the center to the end of the channel, reaching the lower wall in the stretching case. Conversely, in the shrinking case, an opposite trend is observed (refer to Fig. 11). Likewise, an increase in the thermal radiation parameter ${\Gamma }_{d}$ leads to a temperature rise from the upper wall towards the center of the channel in the stretching case. However, only minor changes in temperature are observed from the center to the lower wall of the channel. On the other hand, in the shrinking case, the opposite effect is observed (see Fig. 12). The contour plot of velocity profile for hybrid Casson nanofluids into channel shown in Fig. 13.

A comparison of present results with existing literature is given in Tables 3, 4, 5, 6 and 7, give heat transfer rate and skin friction results.

**Table 7:** Variation results for ${N}_{b},{N}_{t},Pr,, {\Gamma }_{d}, Ec, {\phi }_{p1},{\phi }_{p2}$ by fixing $M=0,{\beta }_{c}=0.5, Re=4.$

${N}_{b}$	${N}_{t}$	$Pr$	$Ec$	${\Gamma }_{d}$	${\phi }_{p1}$	${\phi }_{p2}$	Stretching case		Shrinking case	
							$|\theta ^{\prime}(-3)|$	$|\theta ^{\prime}(3)|$	$|\theta ^{\prime}(-3)|$	$|\theta ^{\prime}(3)|$
0.5	0.1	6.2	0.1	5	1%	1%	0.109478	0.109478	0.194933	0.194933
0.6							0.130976	0.130976	0.147033	0.147033
0.7							0.150745	0.150745	0.100861	0.100861
0.8							0.168785	0.168785	0.056418	0.056416
0.1	0.5						0.021126	0.021126	0.375694	0.375694
	0.6						0.016438	0.016438	0.384782	0.384782
	0.7						0.011655	0.011655	0.393966	0.393966
	0.8						0.006775	0.006779	0.403246	0.403246
0.1	0.1	3.2					0.094352	0.094355	0.392562	0.392562
		3.7					0.077668	0.077664	0.380398	0.380398
		4.2					0.064956	0.064956	0.371137	0.371175
		4.7					0.054949	0.054945	0.363834	0.363834
		6.2	0.1	5			0.034612	0.034614	0.349007	0.349007
				6			0.045607	0.045606	0.357023	0.357023
				7			0.056602	0.056607	0.365039	0.365039
				8			0.067597	0.067599	0.373056	0.373056
			0.5	6			0.143399	0.143399	0.472311	0.472311
			0.6				0.187902	0.187902	0.503137	0.503137
			0.7				0.232405	0.232405	0.533963	0.533963
			0.8				0.276908	0.276908	0.564789	0.564789
			0.5		1%		0.034612	0.034614	0.349007	0.349007
					2%		0.035448	0.035447	0.351725	0.351725
					3%		0.036205	0.036205	0.354327	0.354327
					4%		0.036884	0.036888	0.356822	0.356822
					1%	1%	0.034612	0.034612	0.349007	0.349007
						2%	0.038634	0.038634	0.356585	0.356834
						3%	0.042231	0.042231	0.363135	0.363158
						4%	0.045471	0.045471	0.368869	0.368896

## Conclusion

We consider the physical model of two-dimensional Casson hybrid nanofluid flow under the effects of magnetic field and thermal radiation inside the channel. Following are the key findings of the current study.

1. It can be observed that as the Reynolds number $Re$ increases, the velocity decreases in the center of the channel but increases near the bottom and upper walls in the case of wall contraction.
2. It is seen that in the stretching case, the temperature gradually increases from the upper wall towards the center of the channel, but decreases near the lower wall.
3. It is evident that the hybrid Casson nanofluids exhibit opposite behaviors in the stretching and shrinking cases near the upper and lower walls of the channel.
4. It is also observed that in the stretching case, increasing the values of the Casson parameter leads to a rise in both shear stress and heat transfer rate for both plates of the channel. However, the results contradict this trend in the shrinking case.
5. Increase in the thermal radiation parameter ${\Gamma }_{d}$ leads to a temperature rise from the upper wall towards the center of the channel in the stretching case.

## Data Availability

The datasets used and/or analysed during the current study available from the corresponding author on reasonable request.
